# Progression of sleep disturbances in Parkinson’s disease: a 5-year longitudinal study

**DOI:** 10.1007/s00415-020-10140-x

**Published:** 2020-08-17

**Authors:** Zheyu Xu, Kirstie N. Anderson, Seyed Ehsan Saffari, Rachael A. Lawson, K. Ray Chaudhuri, David Brooks, Nicola Pavese

**Affiliations:** 1 Department of Neurology, National Neuroscience Institute, Singapore, Singapore; 2 Translational and Clinical Research Institute, Newcastle University, Newcastle upon Tyne, UK; 3 Centre for Quantitative Medicine, Duke-National University of Singapore Medical School, Singapore, Singapore; 4 Parkinson Foundation International Centre of Excellence at Kings’ College Hospital, Denmark Hill, London, UK; 5 Institute of Psychiatry, Psychology and Neuroscience at King’s College London, London, UK; 6 Department of Nuclear Medicine and PET Centre, Aarhus University Hospital, Aarhus, Denmark; 7 Clinical Ageing Research Unit, Newcastle University, Campus for Ageing and Vitality, Westgate Road, Newcastle upon Tyne, NE4 5PL UK

**Keywords:** Parkinson’s disease, Sleep, Insomnia, RBD, EDS, PPMI

## Abstract

**Background:**

Sleep disorders can occur in early Parkinson’s disease (PD). However, the relationship between different sleep disturbances and their longitudinal evolution has not been fully explored.

**Objective:**

To describe the frequency, coexistence, and longitudinal change in excessive daytime sleepiness (EDS), insomnia, and probable REM sleep behavior disorder (pRBD) in early PD.

**Methods:**

Data were obtained from the Parkinson’s Progression Markers Initiative (PPMI). EDS, insomnia, and pRBD were defined using the Epworth Sleepiness Scale, MDS-UPDRS Part I sub-item 1.7, and RBD screening questionnaire.

**Results:**

218 PD subjects and 102 controls completed 5 years of follow-up. At baseline, 69 (31.7%) PD subjects reported one type of sleep disturbance, 25 (11.5%) reported two types of sleep disturbances, and three (1.4%) reported all three types of sleep disturbances. At 5 years, the number of PD subjects reporting one, two, and three types of sleep disturbances was 85 (39.0%), 51 (23.4%), and 16 (7.3%), respectively. Only 41(18.8%) patients were taking sleep medications. The largest increase in frequency was seen in insomnia (44.5%), followed by EDS (32.1%) and pRBD (31.2%). Insomnia was the most common sleep problem at any time over the 5-year follow-up. The frequency of sleep disturbances in HCs remained stable.

**Conclusions:**

There is a progressive increase in the frequency of sleep disturbances in PD, with the number of subjects reporting multiple sleep disturbances increasing over time. Relatively a few patients reported multiple sleep disturbances, suggesting that they can have different pathogenesis. A large number of patients were not treated for their sleep disturbances.

**Electronic supplementary material:**

The online version of this article (10.1007/s00415-020-10140-x) contains supplementary material, which is available to authorized users.

## Introduction

Sleep disturbances are common in Parkinson’s Disease (PD) and are recognized to be present in the early stages of disease [1]. A broad range of sleep disorders are seen [1] with potential interactions existing between the different disorders. However, the relationship between these sleep disturbances remains to be fully investigated, as they have mostly been studied in isolation, in the context of a broad range of non-motor symptoms, in cross-sectional or in short-longitudinal cohorts.

Previously, a cross-sectional study by Suzuki and colleagues had investigated excessive daytime sleepiness (EDS), probable REM sleep behavior disorder (pRBD), and other PD-related sleep problems defined using the PD sleep scale (PDSS-2) in a cross-sectional cohort of PD patients in advanced disease. In that cohort, 20% of patients reported two types of sleep disturbances and 12.5% reported all three types of sleep disturbances, with significant overlap identified in the three sleep-related symptoms [2]. However, the PDSS-2 questionnaire itself has components with overlap with the reporting of EDS and pRBD, and does not allow the complaints of insomnia to be studied separately. Furthermore, it is unknown whether overlap observed between these commonly reported sleep-related symptoms in advanced disease is also observed in early PD.

The aim of this study was to examine the frequency, coexistence, and longitudinal changes over a 5-year period of the commonly reported sleep disturbances of EDS, insomnia, and pRBD in a large cohort of initially drug-naïve PD subjects. EDS, insomnia, and pRBD were defined using validated questionnaires to capture the patients’ subjective sleep complaints as reported in clinical settings. Findings were compared with healthy controls (HCs). The long duration of follow-up will help better understand the emergence and the relationship of the various sleep disturbances in early PD.

## Methods

### Study population

PD subjects and the HCs were identified from the Parkinson’s Progression Markers (PPMI) Initiative database. The PPMI is an ongoing longitudinal, multicentre observational clinical study of early PD patients with a clinical disease duration of less than 2 years who were initially treatment naïve. The diagnosis of PD had to be supported by in vivo evidence of nigrostriatal dopaminergic dysfunction by DAT SPECT. Control subjects recruited into the study had to be 30 years or older and not to have a first degree relative diagnosed with PD. The full list of inclusion and exclusion criteria can be found at the PPMI website (https://www.ppmi-info.org/). Each study site obtained local ethical approval.

For our analysis, we identified PD subjects in the PPMI database who had completed 5 years of follow-up. For these subjects, we downloaded baseline assessments and annual follow-up visits. When follow-up evaluations were unavailable, results were obtained from evaluations performed in the preceding or successive 6-month period. Since most patients started anti-parkinsonian medications between baseline and 2 years, we decided to perform the first follow-up evaluation relative to 2 years.

We also extracted data from HCs recruited in the PPMI study with 5-year follow-up. The RBD Screening Questionnaire (RBDSQ) was one of the battery of tests performed to characterize both PD subjects and HCs after inclusion into the PPMI study. As the presence of REM sleep behavior disorder (RBD) is now known to be strongly associated with future neurodegeneration, HCs who screened positive for RBD at any time point during this 5-year period were excluded from further analyses in our study.

Data were downloaded on 2nd November 2017.

### Demographics and assessments:

The sleep disturbances evaluated include EDS, insomnia, and RBD. EDS was evaluated using the Epworth Sleepiness Scale [3] where subjects self-report their likelihood of falling asleep in the preceding month with scores of ≥ 10 indicating EDS [4].

Insomnia complaints were evaluated using sub-item 1.7 of the Movement Disorders Society-Unified Parkinson’s Disease Rating Scale (MDS-UPDRS) [5]. Participants were asked: “Over the past week, have you had trouble going to sleep at night or staying asleep throughout the night? Consider how rested you felt after waking up in the morning.” Clinically relevant insomnia was defined as a MDS-UPDRS sub-item 1.7 score ≥ 2, which was previously used [6] and shown to have good correlation with other insomnia rating scales [7]. The presence of RBD was assessed using the RBDSQ [8]. Subjects were classified as positive for RBD if they scored > 5 [9]. However, since RBD was not confirmed by polysomnography, we have used the term probable RBD (pRBD) in this study.

Demographic and clinical data including PD medication and sleep-related medication use were obtained from the medication logs. The total daily Levodopa equivalent daily dose (LEDD) was calculated using the established methods [10]. Clinical motor assessments performed included the Hoehn and Yahr (H&Y) stage [11] and the MDS-UPDRS Part III [5]. Cognition was evaluated using the Montreal Cognitive Assessment (MOCA) [12].

Statistical analysis was performed using SPSS Version 24 for Windows (Armonk, NY:IBM Corp). Baseline demographics and clinical characteristics were compared between PD and HC groups using Chi-square or Fisher’s exact test for categorical variables and two-sample test and Mann–Whitney *U* test for continuous variables. Similar statistical tests were conducted to compare PD subjects with no sleep disturbance and at least one sleep disturbance.

The number of PD patients reporting multiple types of sleep disturbances (two and three) increased over 5 years. We selected data at the 5-year mark to investigate the association between demographics and clinical features (including age, LEDD, medication class of levodopa replacement therapy used, and MDS-UPDRS Part III scores) and number of reported sleep disturbances (none, one, two, or three) using Chi-Square or Fisher’s exact test and analysis of variance and Kruskal–Wallis test for categorical and continuous variables, respectively. Post hoc pairwise comparisons (versus “No reported sleep disturbance” group as reference) were made using two-sided two-sample *t* test and Mann–Whitney test for continuous variables and Chi-square or Fisher’s exact test for categorical variables. Similarly, the association between demographics and clinical variables PD subjects with no reported sleep disturbances, insomnia only, pRBD only, and EDS only at 5 years were also explored using the above-mentioned statistical methods. Bonferroni adjustment was used to correct for multiple testing. Statistical significance level was set at < 0.05.

## Results

### Analysis of PPMI database and subject characteristics:

Baseline clinical assessments were available for 423 PD subjects. Out of which, 218 completed both baseline and 5-year follow-up and were included in the analysis, although some had missing data for intermediate assessments. Therefore, we had 202 subjects who completed evaluations for 2-year follow-up, 211 for 3-year follow-up, and 210 for 4-year follow-up. The clinical characteristics of these patients, except for disease duration, were not statistically different from those of the 205 patients excluded for the lack of 5-year follow-up (Supplementary Table 1).

Out of 196 HCs with available baseline clinical assessments, 127 had completed evaluations at 5 years. Of those, 25 HCs were excluded as their RBDSQ scores would suggest the presence of pRBD. In total, 102 HCs were included: 100 had evaluations for 2-year follow-up and 98 had evaluations for 3-year and 4-year follow-ups.

Except gender distribution (PD group had a higher proportion of male subjects compared to the HC group: 69% vs 57%, *p* = 0.05), PD subjects and HCs were comparable in terms of baseline demographic variables and use of sleep-related medications. The mean LEDD was 282 ± 233 mg, 397 ± 294 mg, 498 ± 289 mg, and 585 ± 3 36 mg at the 2-, 3-, 4-, and 5-year time points, respectively (Table 1).

**Table 1:** Baseline clinical and demographic features of Parkinson’s disease (PD) and healthy controls (HCs)

	Baseline information of PD subjects n = 218	Baseline information of HCs n = 102	*p* value
N (male/female) %	(150/68) (68.8/31.2)	(58/44); (56.9/43.1)	0.050^a^
Age (years)	60.9 ± 9.4 (min 33, max 84)	59.2 ± 11.1 (min 31, max 81)	0.268^b^
Disease duration (years)	0.59 ± 0.57	NA	
Body mass index	27.1 ± 4.5 (min 18.7, max 43.8)	26.0 ± 4.3 (min 17.5, max 42.3)	0.528^b^
MOCA	27.1 ± 2.3	28.1 ± 1.0	0.001^b^
H & Y staging:			< 0.001^a^
Stage 1	105 (48.1%)	1 (1.0%)	
Stage 2	111 (50.9%)	0 (0.0%)	
Stage 3	2 (0.9%)	0 (0.0%)	
MDS-UPDRS			
Part I	5.35 ± 3.84	2.75 ± 2.98	< 0.001^b^
Part II	5.69 ± 4.19	0.30 ± 0.79	< 0.001^b^
Part III	20.9 ± 8.3	1.23 ± 2.17	< 0.001^b^
Use of sleep-related medications n (%)	16 (7.3%)	9(8.9%)	0.812^a^

Figures are mean ± SD unless otherwise indicated

*H and Y Staging* Hoehn and Yahr Staging, *MDS-UPDRS* Movement Disorders Society–Unified Parkinson’s Disease Rating Scale, *NA* not applicable, *PIGD* Postural Instability and Gait Disorder

^a^Chi-square test

^b^Mann–Whitney *U* test figure

At baseline, 16 (7.3%) PD subjects were using medications for sleep which increased to 41 (18.8%) PD subjects after 5 years (full details in Supplementary Table 2). In contrast, nine (8.9%) HCs were using medications for sleep at baseline which increased to 12 (11.8%) after 5 years.

### Frequency of individual sleep disturbances over the 5-year period

Insomnia was the most common sleep problem reported by our PD patients at any assessment over the 5-year follow-up, followed by pRBD, and EDS. The comparison between PD patients and HCs showed that the proportion of PD and HCs reporting insomnia was not statistically different at baseline and 2-year time points. However, significantly more PD subjects reported insomnia compared to controls at years 3, 4, and 5 (Table 2). Similarly, the proportion of PD and HCs reporting EDS was not statistically different at baseline (*p* = 0.06), but a significantly higher proportion of PD subjects (compared to HCs) reported EDS at all other time points (*p* < 0.05) (Table 2).

**Table 2:** Number and percentage of Parkinson’s disease (PD) and healthy controls (HCs) reporting the presence of insomnia and excessive daytime sleepiness across a 5-year period with between-group comparisons made between the PD and HCs

Time point	Presence of insomnia symptoms			Presence of excessive daytime sleepiness			Presence of REM sleep behavior disorder	
	PD	HCs	*p* value*	PD	HCs	*p* value*	PD	HCs
Baseline	45 (20.6)	15 (14.7)	0.223	37 (17)	9 (8.8)	0.060	46 (21.1)	NA
2 years	64 (31.5)	21 (21)	0.058	47 (23.3)	11 (11)	**0.013**	59 (29.1)	NA
3 years	73 (34.4)	20 (20.6)	**0.016**	59 (27.8)	11 (11.2)	**0.001**	68 (32.2)	NA
4 years	90 (42.7)	17 (17.4)	**0.001**	60 (28.6)	13 (13.3)	**0.004**	67 (31.9)	NA
5 years	97 (44.5)	18 (17.7)	**0.001**	70 (32.1)	10 (9.8)	**0.001**	68 (31.2)	NA

*NA* not applicable

^*^Chi-square test. Significant differences highlighted in bold

### Changes in the frequency of individual sleep disturbances over the 5-year period

In PD patients, the largest increase in frequency over 5 years was seen with insomnia, followed by EDS and then pRBD (Table 2).

At baseline, 45 (20.6%) PD subjects reported insomnia which increased to 97 (44.5%) at 5 years (*p* < 0.001). At baseline, 37 (17.0%) PD subjects reported EDS which increased to 70 (32.1%) at 5 years (*p* < 0.001). At baseline, 46 (21.1%) PD subjects reported pRBD, which increased to 68 (31.2%) at 5 years (*p* = 0.005). In HCs, insomnia and EDS did not change significantly over time (Table 2). At baseline, 15 (14.7%) HCs reported insomnia which increased to 18 (17.7%) at 5 years (*p* = 0.405). At baseline, nine (8.8%) HCs reported EDS which increased to ten (9.8%) after 5 years (*p* = 0.763) (Table 2).

### Coexistence of sleep disturbances in individual subjects

**Fig. 1:**
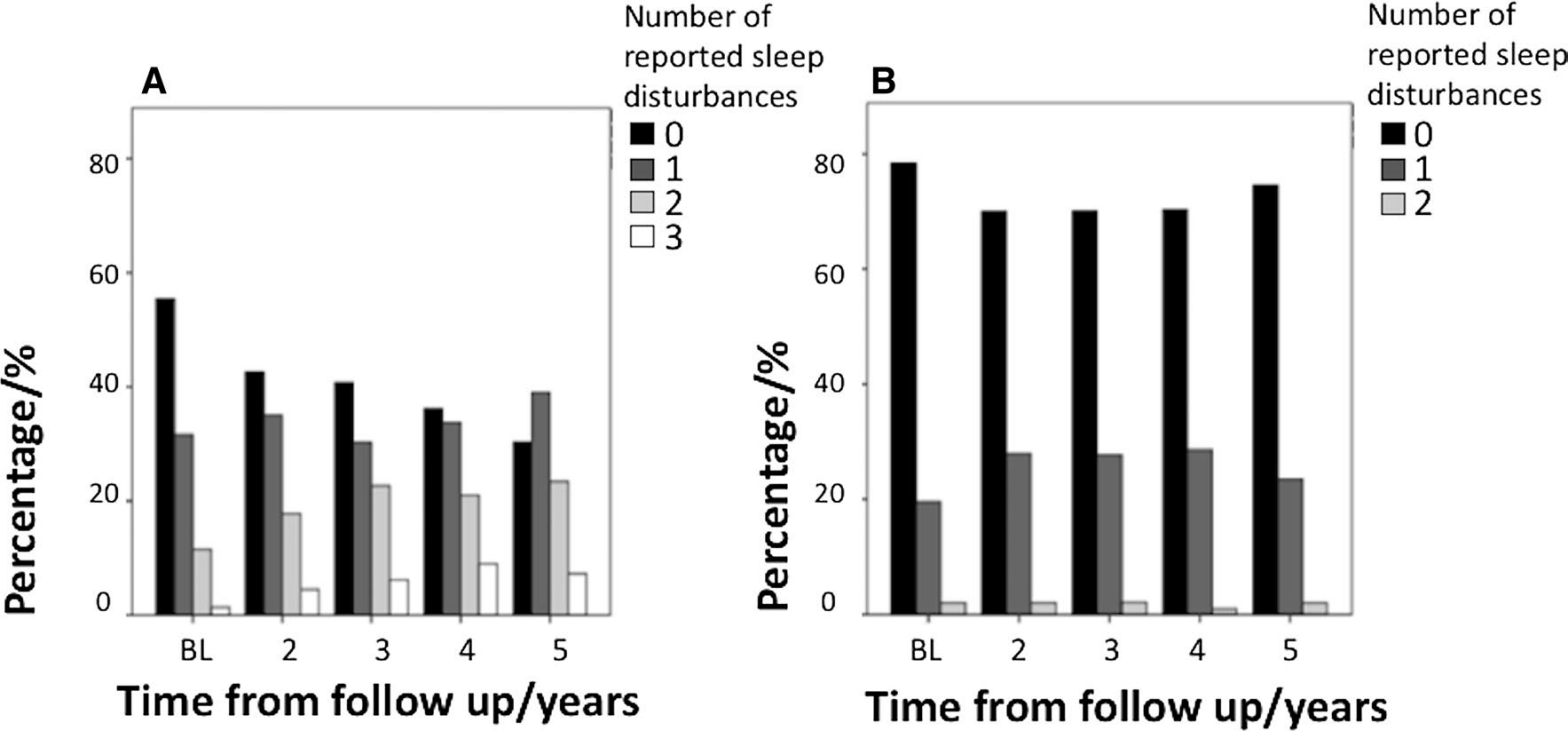
Diagram showing the percentage of subjects reporting none, one, two, or three sleep disturbances in Parkinson’s disease (PD) (**a**) and healthy controls (HCs) (**b**)

The percentage of PD subjects reporting multiple sleep disturbances increased over time, but this was not the case in the HCs (Fig. 1).

### PD subjects

**Fig. 2:**
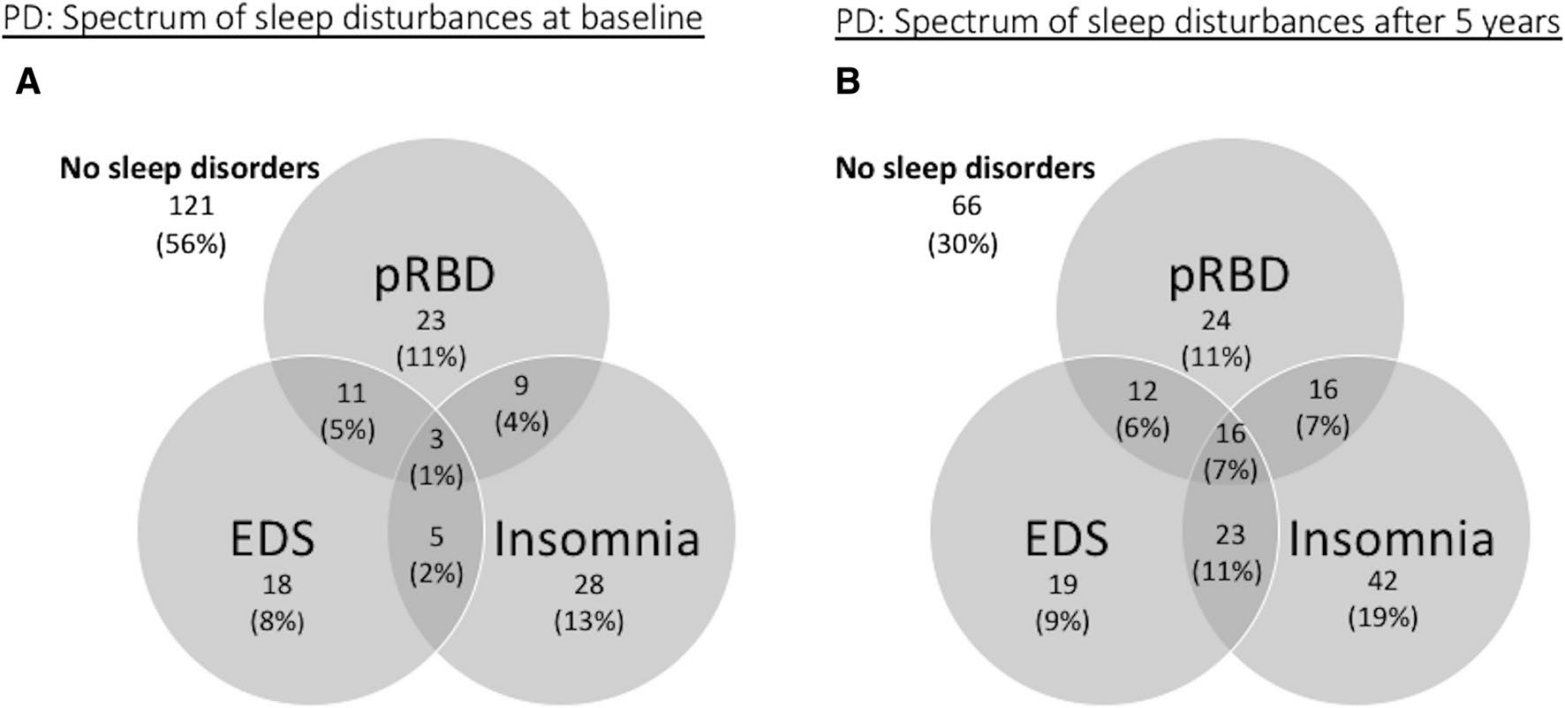
Venn diagram showing the spectrum of sleep disturbances in the Parkinson’s disease (PD) group at baseline (**a**) and 5 years (**b**)

At baseline, 121 (55.5%) PD patients did not report any sleep disturbances. Sixty-nine (31.7%) PD patients reported one type of sleep disturbance: 28 (12.8%) reported insomnia only, 23 (10.6%) reported pRBD only, and 18 (8.3%) EDS only. Twenty-five (11.5%) PD patients reported two types of sleep disturbances: 11 (5.0%) reported both EDS and pRBD, nine (4.1%) reported both insomnia and pRBD, and five (2.3%) reported both insomnia and EDS. Three (1.4%) PD patients reported all three types of sleep disturbances (Fig. 2a).

At 5 years, 66 (30.3%) PD patients did not report any sleep disturbances. Eighty-five (39.0%) PD patients reported one sleep type of disturbance: 42 (19%) reported insomnia only, 24 (11.0%) reported pRBD only, and 19 (8.7%) reported EDS only. Fifty-one (23.4%) PD patients reported two types of sleep disturbances; 23 (10.6%) reported both insomnia and EDS; 16 (7.3%) reported both insomnia and pRBD; and 12 (5.5%) reported both pRBD and EDS. Sixteen (7.3%) PD patients reported all three types of sleep disturbances (Fig. 2b).

#### Healthy controls

At baseline, 20 (19.6%) HCs reported one type of sleep disturbance, 13 (12.8%) insomnia, and 7 (6.9%) EDS). Two (2%) HCs reported two types of sleep disturbances (EDS and insomnia). At 5 years, 24 (23.5%) reported one type of sleep disturbance, 16 (15.7%) insomnia, and 8 (7.8%) EDS, and two (2.0%) HCs reported two types of sleep disturbances.

### Comparisons among PD subjects with and without sleep disturbances at 5 years

PD subjects were initially divided in two groups based on the absence or presence of sleep disturbance (insomnia or EDS or pRBD or any combination of the three sleep complaints) at 5 years. Statistically significant differences in LEDD at the 5-year follow-up were found between the two groups with higher median LEDD reported in subjects reporting sleep disturbances compared to subjects who reported no sleep disturbances [600 (400–800) vs. 455 (300–650) mg, *p* = 0.009].

The two groups were not statistically different in patient age (*p* = 0.655), gender (*p* = 0.443), MOCA (*p* = 0.500), MDS-UPDRS Part III scores (*p* = 0.456), medication status (no medications, dopamine agonists only, levodopa only, and both dopamine agonists and levodopa) (*p* = 0.114), and H & Y stage (*p* = 0.331) (Supplementary Table 3).

Pairwise group comparisons were then made between PD subjects who reported no sleep disturbances and PD subjects who reported insomnia only, EDS only, and pRBD only. PD subjects who reported pRBD were more likely to be male compared to PD subjects who reported no sleep disturbances (87.5% versus 65.1%, *p* = 0.04). PD subjects who reported insomnia had a higher LEDD compared to PD subject who reported no sleep disturbances (639.7 ± 353.5 mg versus 495.2 ± 284.4 mg, *p* = 0.033). All other group-wise comparisons including age, gender, BMI, MOCA, H & Y stage, MDS-UPDRS Part III scores, medication status, and LEDD were not statistically significant (Supplementary Table 4).

Finally, PD patients were grouped into whether they reported none, one, two, or three types of sleep disturbances at 5 years. Statistically significant differences in LEDD at the 5-year follow-up were found between groups using the Kruskal–Wallis test (*χ*^2^ = 11.54, *p* = 0.009). Post hoc analysis found that subjects reporting three types of sleep disturbances had a higher median LEDD (800 mg) compared to subjects who reported no sleep disturbances (455 mg) (*p* = 0.006). Subjects reporting only one sleep disturbance with a median LEDD of 512.5 mg and subjects reporting two sleep disturbances only with a median LEDD of 600 mg were not statistically different from subjects with no sleep disturbances. No other statistically significant differences were found.

The four groups were not significantly different in patient age (*p* = 0.413), gender (p = 0.510), MOCA (*p* = 0.289), and MDS-UPDRS Part III scores (*p* = 0.548). No group differences in medication status (no medications, dopamine agonists only, levodopa only, and both dopamine agonists and levodopa) were found between the patient groups using the Chi-square test (*p* = 0.562).

## Discussion

Our study evaluated the longitudinal progression of three common sleep disturbances (EDS, insomnia, and RBD) and their interactions over a 5-year period in early PD. We found a progressive increase in the frequency of the different types of sleep disturbances. The greatest increase in frequency was observed for insomnia, followed by EDS and pRBD. Insomnia was also the most common sleep problem reported at any time over the 5-year follow-up. The percentage of patients with at least one type of sleep disturbance increased from 46% at baseline to 70% after 5 years. The percentage of patients with multiple sleep disturbances also increased over time, but remained relatively small. Interestingly, one-third of PD patients continued to remain free of sleep disturbances when assessed using our methodology. In contrast, we found that the frequency of sleep disturbances (EDS and insomnia) in HCs remained stable over time.

The novel aspect of our study is that we have assessed the coexistence of EDS, pRBD, and insomnia and their progression in individual patients over 5 years. In our longitudinal assessment, we found a progressive increase in the number of patients reporting multiple sleep disturbances. However, the proportion of these patients remained small. The low percentage of patients with multiple sleep problems suggests that the pathophysiology of the different sleep disturbances in early PD might be due to different mechanisms that attenuate or influence the co-expression of other sleep disturbances.

EDS in PD patients is multifactorial, but could potentially be caused by insomnia and poor sleep. However, in our cohort, only a minority of PD subjects had co-existing insomnia and EDS at baseline, with the proportion of patients with these two disturbances increasing over time but remaining low at 11%. Similarly, only 2% of HCs reported co-existing insomnia and EDS at baseline, which remained unchanged after 5 years. These findings would suggest that insomnia and EDS are not related in the majority of PD patients. We recently reported that EDS was associated with reduced postsynaptic dopaminergic receptor availability measured with [11C]-PHNO PET, but that was not the case for sleep-onset and sleep-maintenance insomnia [13]. However, we acknowledge that EDS could potentially be due to another undiagnosed primary sleep disorder such as obstructive sleep apnoea and Restless Legs syndrome, which were not assessed in this study. Similarly, the presence of comorbid mental health problems is likely to be relevant in an unknown proportion of patients with sleep-related complaints.

It is notable that one-third of our patients continued to remain free of sleep complaints. Although the use of questionnaires might have under-estimated the occurrence of sleep disorders, cross-sectional studies using polysomnography have also identified a proportion of patients (3.5–20%) free of sleep disturbances [14–16], possibly representing a milder disease phenotype. PD subjects with no reported sleep complaints after 5 years did not differ from PD subjects who reported any sleep disturbance, except that higher LEDDs were observed in subjects who reported any sleep disturbance or insomnia only.

We have also evaluated the longitudinal progression of the individual sleep disturbances over the 5-year period. Only a few studies have examined the longitudinal change of different sleep disturbances in early PD often with inconsistent findings. One study performed over 2 years did not find any change in the reported frequencies of daytime somnolence, insomnia, and dream enactment in a cohort of 97 newly diagnosed PD patients [17]. However, no control population was investigated and the coexistence of different sleep problems in individual patients was not assessed. The same cohort of patients was reassessed at 4-year follow-up and, while there was a significant increase in frequency of EDS, the frequencies of insomnia and dream enactment were similar to baseline [18]. In another study, which used a self-designed sleep questionnaire, occurrence of insomnia in general remained stable from baseline through a 5-year period [17–19]. Using the PPMI database over a shorter duration, Simuni and colleagues did not find any significant increase in reported frequency of EDS, insomnia, and pRBD over 2 years [20], while Amara and colleagues reported an increased frequency of EDS and pRBD over 3 years, but did not study insomnia [21]. Finally, two studies reported an increase in the frequency of pRBD in early PD cohorts [22, 23].

We found that insomnia was the most common sleep problem reported at any time over the 5-year follow-up. This finding is consistent with cross-sectional studies in advanced PD that also found insomnia to be the most commonly reported sleep disturbance [14, 24, 25]. However, we found that the frequency of insomnia in PD patients was significantly higher than in HCs only after 3 years of follow-up. Insomnia also had the greatest increase in frequency that rose steadily during the 5 years. This is consistent with polysomnographic studies showing progressive sleep destructuring with disease progression [26]. Taken together, these results would suggest that development of insomnia in PD mainly occurs in mid- to advanced stages of disease. However, our study design did not allow us to make the distinction between sleep-onset and sleep-maintenance insomnia. A previous study in early PD has suggested that the prevalence of sleep-maintenance insomnia increases over time, but that of onset insomnia decreases over time [19].

We also found that pRBD frequency increased from 21% at baseline to 31% after 5 years, which is consistent with findings from two previous studies in smaller cohorts of early PD patients [22, 23]. However, in comparison to these studies [22, 23], we report a slower increase in pRBD frequency of approximately 2% per year. This is probably due to the increasing proportion of subjects treated for RBD in our study over time.

We then assessed the possible effects of dopamine replacement drugs on sleep disorders. Our findings suggest that the overall impact of LEDD on the total number of reported sleep disorders was small. PD patients reporting three types of sleep disturbances had a higher median LEDD compared to subjects without sleep disturbances. However, no differences were observed in the other pairwise comparisons where subjects reported fewer (zero, one, or two) types of sleep disturbances, although there was a trend for higher LEDDs to be associated with increasing number of reported sleep disturbances. This observed trend could reflect more severe disease being associated with a greater number of sleep disturbances. However, this is also complicated by the use of dopamine agonists in younger patients in milder disease, which is associated with excessive somnolence. In other studies investigating isolated sleep symptoms in early PD, LEDDs have been reported to be higher in EDS [19] but not different in insomnia [19] and RBD [23].

The previous studies in early PD have not reported differences in MDS-UPDRS Part III scores in subjects reporting EDS [27, 28], insomnia [19], and RBD [23, 29]^.^ Consistent with the previous studies, we did not find differences in age and number of sleep disturbances [21, 27, 28], insomnia [19], and RBD [23, 29].

Interestingly, despite finding a progressive increase in the frequency of the different types of sleep disturbances in our patients and, at the 5-year visit, 70% of the patients were complaining of sleep problems, there was only a small increase in the proportion of patients taking sleep medications at 5-year follow-up compared to baseline. Although it is possible that not all sleep complaints are severe enough to require medical therapy, our findings indicate that a large number of patients with PD do not receive the appropriate clinical attention for their sleep disorder.

The major strength of the study is the large number of well-characterized early PD subjects who were assessed at multiple time points. Additionally, the high retention rate allowed for the longitudinal analysis of sleep characteristics. Thus, our findings are based on assessments that can be performed as part of the routine standard of care and realistically reflect the patients’ subjective sleep complaints reported in clinical settings. There are, however, a number of limitations. While the MDS-UPDRS sub-item 1.7 was previously used [6] to screen clinically relevant insomnia and shown to have good correlation with other insomnia rating scales [7], we did not perform any objective measures of EDS or RBD. Therefore, the real frequency of sleep symptoms and other undiagnosed primary sleep disorders in our patients could be under-estimated in the absence of polysomnography. However, we wanted to capture the prevalence of sleep problems with simple tools that could reflect the complaints reported by the patients and be used in routine clinical settings. There was a higher proportion of male subjects in the PD group relative to the HCs group, which could have either over-estimated or under-estimated the relative expression of the different sleep disturbances. In the general population, the male gender was associated with a lower reporting of insomnia complaints [30, 31] but higher reporting of dream enactment behaviors [32]. In the PD population, gender has been shown not to influence reporting of RBD using the RBDSQ [33], but more frequent reporting of EDS [27, 34, 35]. Overall, the effect of gender on sleep complaints could have under-estimated the frequency of increased insomnia complaints in the PD group relative to the HC group, but over-estimated the relative increased frequency of EDS and RBD in our cohort.

In conclusion, we present 5-year longitudinal data from the PPMI cohort and show that the frequency of insomnia, EDS, and pRBD all increase over time in early PD, with insomnia frequency increasing most rapidly. At the individual level, the profile of sleep disturbances reported was variable, necessitating careful clinical assessment to identify the underlying sleep complaints, as they have different treatments. Although sleep disturbances are common, one-third of the PPMI cohort remained free of sleep complaints after 7 years of disease. Finally, the small number of patients with multiple types of sleep disturbances suggests that the different disturbances have different pathogenesis.

## Electronic supplementary material

Below is the link to the electronic supplementary material.

Supplementary file1 (DOCX 16 kb)

Supplementary file2 (DOCX 14 kb)

Supplementary file3 (DOCX 15 kb)

Supplementary file4 (DOCX 15 kb)

## Data Availability

Source data are available on the PPMI website.
