# Supplementary material for: Progression of sleep disturbances in Parkinson’s disease: a 5-year longitudinal study
Source: J Neurol. 2020 Aug 17;268(1):312–20. doi: 10.1007/s00415-020-10140-x (PMC7815601; doi:10.1007/s00415-020-10140-x)
Supplement: Supplementary file 1 — Supplementary file1 (DOCX 16 kb) [file 415_2020_10140_MOESM1_ESM.docx]

**Supplementary Table 1:** Baseline clinical and demographic features of included and excluded Parkinson ‘s Disease (PD) subjects

|  | **Baseline Information of included PD subjects**  **n=218** | **Baseline Information of excluded PD subjects**  **n= 205** | **p value** |
| --- | --- | --- | --- |
| N (male/female)  % | (150/68)  (68.8/31.2) | (127/78)  (62.0/38.0) | 0.138^#^ |
| Age (years) | 60.9 + 9.4 | 61.5 + 10.1 | 0.335^ |
| Disease duration (years) | 0.59 + 0.57 | 0.51 + 0.53 | **0.046^** |
| Body Mass Index | 27.1 + 4.5 | 27.2 + 4.8 | 0.586^ |
| MOCA | 27.1 + 2.3 | 27.2 + 2.3 | 0.379^ |
|  |  |  |  |
| H & Y Staging: |  |  | <0.055^#^ |
| Stage 1 | 105 (48.1%) | 80 (39.0%) |  |
| Stage 2 | 111 (50.9%) | 125 (61.0%) |  |
| Stage 3 | 2 (0.9%) | 0 (0.0%) |  |
|  |  |  |  |
| MDS-UPDRS: |  |  |  |
| Part I | 5.35 +3.84 | 5.81 + 4.28 | 0.418^ |
| Part II | 5.69 + 4.19 | 6.14 + 4.19 | 0.146^ |
| Part III  Sleep disorders: | 20.9 + 8.3 | 20.9 + 9.40 | 0.766^ |
| Insomnia  EDS  pRBD | 45(20.6%)  37 (17.0%)  46 (21.1%) | 54 (26.3%)  28 (13.7%)  48 (23.4%) | 0.158^#^  0.356^#^  0.378^#^ |
|  |  |  |  |
| Use of sleep-related medications n (%) | 16 (7.3%) | 22(10.7%) | 0.223^#^ |

*Figures are mean ± SD unless otherwise indicated. Statistically significant p values are highlighted in* ***bold****. H and Y Staging = Hoehn and Yahr Staging. MDS-UPDRS = Movement Disorders Society – Unified Parkinson’s Disease Rating Scale. EDS = Excessive daytime sleepiness. pRBD = probable REM sleep behaviour disorder.*

^#^Chi-Square test, ^^^Mann Whitney-U test figure
