# Supplementary material for: Progression of sleep disturbances in Parkinson’s disease: a 5-year longitudinal study
Source: J Neurol. 2020 Aug 17;268(1):312–20. doi: 10.1007/s00415-020-10140-x (PMC7815601; doi:10.1007/s00415-020-10140-x)
Supplement: Supplementary file 2 — Supplementary file2 (DOCX 14 kb) [file 415_2020_10140_MOESM2_ESM.docx]

**Supplementary Table 2**. Sleep related medication use in Parkinson’s Disease (PD ) subjects and Healthy Controls (HCs) at baseline and 5 years.

| **Medication Use at Baseline** | | |
| --- | --- | --- |
|  | **PD**  **n=218** | **HCs**  **n=102** |
| *Use of all sleep-related medications n (%)* | 16(7.3%) | 9(8.8%) |
| Antihistamines n(%) | 1(0.5%) | 0(0.0%) |
| Benzodiazepines n(%) | 7(3.2%)^ | 4(3.9%)^#^ |
| Benzodiazepine receptor agonists n(%) | 4(1.8%) | 3(2.9%) |
| Melatonin n(%) | 2(0.9%) | 1(1.0%) |
| Other supplements n(%) | 0(0.0%) | 1(1.0%) |
| Sedating antidepressants n(%) | 3(1.4%) | 0(0.0%) |
| Stimulants n(%) | 1(0.5%) | 1(1.0%) |
| **Medication Use at 5 years** | | |
|  | **PD**  **n=218** | **HCs**  **n=102** |
| *Use of all sleep-related medications n (%)* | 41(18.8%) | 12(11.8%) |
| Antihistamines n(%) | 0(0.0%) | 4(3.9%) |
| Benzodiazepines n(%) | 13(6.0%)^^^ | 1(1.0%)^#^ |
| Benzodiazepine receptor agonists n(%) | 8(3.7%) | 4(3.9%) |
| Melatonin n(%) | 10(4.6%) | 4(3.9%) |
| Other supplements n(%) | 2(0.9%) | 1(1.0%) |
| Sedating antidepressants n(%) | 15(6.9%) | 1(1.0%) |
| Stimulants n(%) | 2(0.9%) | 0(0.0%) |

Figures indicate the number and percentage of subjects reporting sleep related medication use.

^^^ At baseline, 6/7 PD subjects using benzodiazepines were prescribed clonazepam, with the remaining subject prescribed temazepam. At 5 years, 13/13 subjects using benzodiazepines were prescribed clonazepam.

^#^ At baseline, none of the HCs were using clonazepam. At 5 years, one HC was prescribed clonazepam.
