# Supplementary material for: Progression of sleep disturbances in Parkinson’s disease: a 5-year longitudinal study
Source: J Neurol. 2020 Aug 17;268(1):312–20. doi: 10.1007/s00415-020-10140-x (PMC7815601; doi:10.1007/s00415-020-10140-x)
Supplement: Supplementary file 3 — Supplementary file3 (DOCX 15 kb) [file 415_2020_10140_MOESM3_ESM.docx]

**Supplementary Table 3.** Table comparing clinical variables in Parkinson’s disease (PD) subjects reporting no sleep disturbance after 5 years and PD subjects reporting at least one sleep disturbance after 5 years.

| Variable | PD subjects with no reported sleep disturbance after 5 years  (n=66) | PD subjects reporting at least 1 sleep disturbance after 5 years  (n=152) | p value^*^ |
| --- | --- | --- | --- |
| Female | 23 (34.9)^**^ | 45 (29.6) | 0.443 |
|  |  |  |  |
| Age (years) | 66.4 ± 9.0  (min 61, max 74) | 65.7 ± 9.5  (min 59, max 73) | 0.614 |
|  |  |  |  |
| BMI | 26.1 ± 4.8  (min 23, max 28.8) | 26.9 ± 4.4  (min 23.2, max 29.5) | 0.250 |
|  |  |  |  |
| MOCA | 27.2 ± 2.0 | 26.1 ± 4.2 | **0.048** |
|  |  |  |  |
| H & Y staging:  Stage 1  Stage 2  Stage 3  Stage 4  Stage 5 | 6 (9.1%)  58 (87.9%)  1 (1.5%)  1 (1.5%)  0 (0.0%) | 22 (14.5%)  116 (76.3%)  10 (6.6%)  3 (2.0%)  1 (0.7%) | 0.331 |
|  |  |  |  |
| MDS-UPDRS Part III | 25 ± 10.6 | 26.9 ± 13.3 | 0.307 |
|  |  |  |  |
| Medication status:  Not medicated  Dopamine agonists only  Levodopa only  Dopamine agonists and levodopa | 9 (13.6%)  10 (15.2%)  31 (47.0%)  16 (24.2%) | 8 (5.3%)  16 (10.5%)  83 (54.6%)  45(29.6%) | 0.114 |
|  |  |  |  |
| LEDD/mg | 495.2 ± 284.4  (min 300, max 650) | 623.6 ± 349.1  (min 400, max 800) | **0.009** |
|  |  |  |  |

*Figures are mean + SD unless otherwise indicated.*

^*^ Chi-square test or Fisher’s exact test for categorical variables; Two-sample t test comparing means and Mann-Whitney U test comparing medians for continuous variables

^**^ Categorical variables reported as frequency (%); Continuous variables reported as mean ± standard deviation and median (first quartile-third quartile)

PD=Parkinson’s Disease; BMI=Body Mass Index; H & Y= Hoehn and Yahr; MDS-UPDRS= Movement Disorder’s Society -Unified Parkinson’s Disease Rating Scale; LEDD= Levodopa equivalent daily dose

Statistically significant differences (p<0.05) are highlighted in **bold**.
