# Supplementary material for: Progression of sleep disturbances in Parkinson’s disease: a 5-year longitudinal study
Source: J Neurol. 2020 Aug 17;268(1):312–20. doi: 10.1007/s00415-020-10140-x (PMC7815601; doi:10.1007/s00415-020-10140-x)
Supplement: Supplementary file 4 — Supplementary file4 (DOCX 15 kb) [file 415_2020_10140_MOESM4_ESM.docx]

**Supplementary Table 4.** Table comparing clinical variables in Parkinson’s disease (PD) subjects reporting no sleep disturbance, insomnia only, pRBD only and EDS only at 5 years.

| Variable | No reported sleep disturbance  (n=66)^+^ | Insomnia only  (n =42) | pRBD only  (n=24) | EDS only  (n=19) |
| --- | --- | --- | --- | --- |
| Female | 23 (34.9%) | 16 (38.1%) | 3 (12.5%)^*^ | 5 (26.32%) |
| Age (year) | 66.4 ± 9 | 66 ± 10.7 | 67.4 ± 10.2 | 66.6 ± 9.7 |
| BMI | 26.1 ± 4.8 | 26.6 ± 5.1 | 25.7 ± 2.8 | 28.3 ± 5.6 |
| MOCA | 27.2 ± 2 | 26.1 ± 4 | 26.4 ± 3.2 | 27.1 ± 3 |
|  |  |  |  |  |
| H & Y Staging: |  |  |  |  |
| Stage 1  Stage 2  Stage 3  Stage 4 | 6 (9.1%)  58 (87.9%)  1 (1.5%)  1 (1.5%) | 8 (19.1%)  30 (71.4%)  3 (7.1%)  1 (2.4%) | 2 (8.3%)  19 (79.2%)  3 (12.5%)  0 (0%) | 3 (15.8%)  14 (73.7%)  1 (5.3%)  1 (5.3%) |
|  |  |  |  |  |
| MDS-UPDRS Part III | 25 ± 10.6 | 26.1 ± 11.9 | 29.7 ± 12.6 | 26.2 ± 12.7 |
|  |  |  |  |  |
| Medication status :  Not medicated  Dopamine agonists only  Levodopa only  Dopamine agonists & levodopa | 9 (13.6%)  10 (15.2%)  31 (47.0%)  16 (24.2%) | 3 (7.1%)  3 (7.1%)  24 (57.1%)  12 (28.6%) | 3 (12.5%)  4 (16.7%)  11 (45.8%)  6 (25.0%) | 0 (0%)  3 (15.8%)  10 (52.6%)  6 (31.6%) |
|  |  |  |  |  |
| LEDD/mg | 495.2 ±284.4 | 639.7 ±353.5^*^ | 524.1 ±256.1 | 605.3 ±338.9 |

*Continuous variables are reported as mean + standard deviation, catergorical variables are reported as frequency(percent).*

^*^ Significantly different from “No reported sleep disturbance” group at p < 0.05 level.

Two-sided two-sample t-test and Mann-Whitney test for continuous variables and Chi-square or Fisher’s exact test for categorical variables are conducted for pairwise comparison between the two groups.
